# Ram’s Horn Nail in Lower-Limb Ischemia

**Published:** 2015-07-03

**Authors:** Khosro Barkhordari, Akbar Shafiee

**Affiliations:** Tehran Heart Center, Tehran University of Medical Sciences, Tehran, Iran.

**Keywords:** *Nails*, *Nail disease*, *Lower extremity*, *Ischemia *

A 71-year-old man, candidated for coronary artery bypass graft surgery, was admitted to our center. He was a known case of type II diabetes mellitus, receiving oral hypoglycemic agents, and had controlled hypertension. During physical examination, deformity and gross thickening of the right first toe nail was observed, which had given the nail a claw-like appearance. The surface of the nail was rough, scaly, and olive-colored and it extended to the ventral surface of the toe. A consultant dermatologist diagnosed it as onychogryphosis or ram’s horn nail, which is mostly observed in the elderly and can result from peripheral vascular insufficiency or frequent trauma to the nail. The patient was referred to a dermatology center for further evaluation and appropriate treatment. 

Onychogryphosis of the toenails is mainly due to self-neglect; however, it may also be a feature of chronic ischemia or nail changes associated with diabetes mellitus.^1^^-^^2^ This condition is mostly observed in the elderly and can result from frequent trauma to the nail.^   3 ^

**Figure 1:**
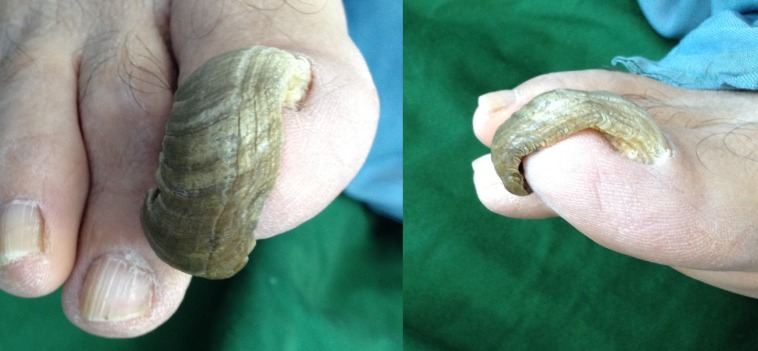
Anterior (left pane) and lateral (right pane) views of the ram’s horn nail

## Notes:

***This paper should be cited as: ***Barkhordari K, Shafiee A. Ram’s Horn Nail in Lower-Limb Ischemia. J The Univ Heart Ctr 2015;10(3):163-164.
